# Revisiting the disproportionate COVID-19 mortality of ethnic minorities in light of the migrant mortality advantage

**DOI:** 10.3389/fpubh.2023.1284041

**Published:** 2023-11-30

**Authors:** Myriam Khlat

**Affiliations:** Unité “Mortalité, santé, épidémiologie”, Institut national d'études démographiques (INED), Aubervilliers, France

**Keywords:** COVID-19, mortality, ethnic groups, migrant mortality advantage, second generations

The COVID-19 pandemic has hardly hit ethnic minorities in most high-income countries, with extensive evidence from the United Kingdom and the United States (1, 2) of excess mortality in those groups. Many studies have categorized individuals based on their self-reported race/ethnicity, not considering place of birth. It needs to be emphasized, however, that ethnic groups are composed of immigrants and their offspring born in the receiving country, and that this inherent heterogeneity must be considered when analyzing mortality, contrary to usual practice.

Ethnicity is a complex concept that encompasses cultural traditions and a shared sense of identity, language, and connections to a geographical region (3). Racial and ethnic health disparities are therefore related to immigration, as individuals' ethnic identities find their roots in their own or their ancestors' country of origin. In the realm of epidemiology, there is a long tradition of studying migrants to assess the effects on health of a change in living environment following migration. In the realm of public health, the persistence of ethnic disparities remains a matter of concern.

Although self-reporting of race/ethnicity has become increasingly common in health research, the country of birth indicator has the advantage of objectivity and stability (4). A focus on self-reported race/ethnicity produces groups whose composition by place of birth varies according to the length of migration history. First-generation immigrants are those individuals residing in a country other than that of their birth, and their children born in the receiving country are called “second generations.” When the history of migration of the group to the receiving country is long, most of the individuals belong to the second or higher generation; when it is short, the share of first-generation immigrants is large.

According to the latest US data, only one-in-ten Black people were immigrants,^1^ while this was the case for 32% of those identifying as Hispanic or Latino,^2^ and for as many as six-in-ten Asian Americans (see text footnote ^1^). In Europe, migration originating from non-European countries mostly developed after the Second World War. Given this chronology, ethnic minority groups comprise relatively large proportions of immigrants, and the native-born members of those groups are mostly children of immigrants. For instance, in the United Kingdom in the early 2010s, 58% of Asians and 53% of Black persons were born abroad.^3^

In the European context, immigrants and their children are likely to have different baseline mortality profiles. Immigrants, particularly those born in non-Western countries, tend to differ from other socioeconomically disadvantaged groups in that they exhibit relatively low mortality from major chronic diseases. This comparative advantage is largely attributable to the selection of healthy individuals into migration, as the healthiest individuals from the countries of origin are both more likely to decide to migrate (self-selection) and to pass the health screenings of the destination country (external selection) (5). Selective factors, which do not apply to certain migrant subgroups such as refugees, interact with the social determinants of health in shaping the health of migrants. In the midst of the COVID-19 crisis, the migrants' health assets were masked by their vulnerability to infection, attributable to their living and working conditions and to the specific barriers they may have faced to access health care. This is strikingly illustrated by a study from France. When a strict lockdown gripped the entire country in the spring of 2020, migrants' death rates overshoot those of the native-born in the most heavily affected regions, whereas at the same time, their mortality advantage was quite visible in the least affected regions (6).

Regarding the children of immigrants, they differ from their parents in that they are not a selected group, which does not necessarily imply that they may not enjoy, at least partly, a mortality advantage. According to the “cultural effects” hypothesis (7), migrants may have more favorable health behaviors than non-migrants due to the cultural norms of their country of origin, and from there it may be expected that their children integrate some of those traditional health behaviors. More generally, children of immigrants could benefit to some extent of an intergenerational transmission of health outcomes across generations (8). However, the existence of a mortality advantage in second-generation migrants has found no support in the literature. A review of recent studies from Europe has even disclosed a cross-generational “reversal” of the mortality advantage in comparison with the natives, with the observation of a systematic mortality disadvantage among second-generation migrants in their early life and adulthood (9, 10).

This generational polarization within ethnic minorities has at least two major implications. First, the reversal of the mortality differential across migrants' generations has to be considered and addressed through public policy. In a number of countries, the share of children of immigrants within the child and youth population is growing rapidly (11). In both the United Kingdom (12) and France (13), second-generation immigrants were found to be better educated than their parents, and in the United Kingdom, they were even found to be better educated than their peers in the majority population. Yet, with the same educational profiles, they were less likely in both countries to find employment and earned lower wages. This may be attributable to discrimination, potentially leading to stress, feelings of hopelessness, and risk-taking behaviors. The excess mortality of second generations in different European countries is an alarming consequence of this enduring disadvantage (10). For laying out sound policies, better insight is needed into the life outcomes of these populations, their behaviors, and their experience of discrimination and barriers in different life spheres. Data collection efforts could be strengthened both through surveys specifically covering immigrants and their children, and through the inclusion of specific questions on parents in general population surveys, allowing second generations to be identified (14).

Second, the distinction between first- and second-generation migrants is all the more important in mortality analyses, as their respective mortality levels may counterbalance each other within ethnic groups. The impact of health crises, particularly when it is not limited to older adults, is also bound to be different in those two subgroups which do not have the same baseline mortality levels. One of the rare studies on COVID-19 that stratified ethnic minorities by birthplace was from Denmark, and the authors reported that during the first year of the pandemic, the odds of 30-day mortality among hospitalized COVID-19 cases was lower among immigrants than among individuals of Danish origin, but that it was not among their descendants (15). During the first COVID-19 wave in France (6), the mortality advantage for migrants from sub-Saharan Africa and North Africa reversed, but the impact on the mortality of children of immigrants could not be assessed as they were indistinguishable from the majority population in the absence of data enabling their identification. Data collection protocols must be devised so that mortality and causes of death for the native-born offspring of immigrants can be assessed. This could be achieved, for instance, with questions on parental places of birth in censuses or surveys that can be linked to death registration data.

## Author contributions

MK: Conceptualization, Writing – original draft, Writing – review & editing.

## Footnotes

^1^See: https://www.pewresearch.org/short-reads/2022/01/27/key-findings-about-black-immigrants-in-the-u-s/.

^2^See: https://www.migrationpolicy.org/article/frequently-requested-statistics-immigrants-and-immigration-united-states.

^3^See: https://www.ethnicity-facts-figures.service.gov.uk/uk-population-by-ethnicity/demographics/people-born-outside-the-uk/latest#place-of-birth-uk-or-non-uk-by-ethnicity.
